# The Efficacy of Local Autologous Platelet-Rich Plasma Prepared by Single and Double Spin Methods in the Treatment of Chronic Ulcer

**DOI:** 10.7759/cureus.61366

**Published:** 2024-05-30

**Authors:** Devesh Dubey, Babita Raghuwanshi

**Affiliations:** 1 Transfusion Medicine, All India Institute of Medical Sciences, Bhopal, IND

**Keywords:** nonhealing ulcers, prp variations, double spin prp, single spin prp, platelet rich plasma, autologous, chronic ulcers, ulcer healing

## Abstract

Background: Chronic nonhealing ulcers present significant challenges in diabetic, dermatological, and surgical patients. Platelet-rich plasma (PRP), enriched with bioactive factors, offers promise for wound healing enhancement. This study evaluates PRP's efficacy, prepared via single and double spin methods in nonhealing chronic ulcers.

Methods: Twenty-two patients aged 18-65 years participated and 100 mL of blood was drawn into citrate phosphate dextrose adenine (CPDA) bags with all aseptic precautions. PRP was prepared by single and double spin methods. Patient serum and 10% calcium gluconate were added to fibrin gel. PRP was injected around the ulcer and then dressed. Dressings were changed on the fifth, 15th, and 20th days with PRP. Evaluation occurred on day 30 using surface area and volume assessments by both methods.

Results: The single spin PRP group and double spin PRP group had 11 patients each with hemoglobin range of 10.79±1.88 to 12.63±2.22 g/dL. Initial lesions (16.27 cm²) significantly reduced to 14.76 cm² after double spin PRP sessions (p=0.005) and Initial lesions (9.87 cm²) significantly reduced to 7.65 cm² after single spin PRP sessions (p=0.005). Platelet count differences between whole blood and PRP were significant (p<0.05).

Conclusions: The single spin PRP method exhibited considerable improvements in healing parameters, showcasing its potential for chronic ulcer management.

## Introduction

Platelet-rich plasma (PRP) has undergone a systematic transition, traversing domains encompassing hematology, dentistry, and sports medicine and subsequently permeating the domains of dermatology, esthetics, and trichology. Despite the proliferation of data disseminated, published, and amassed, a conspicuous paucity of reproducible information prevails. This insufficiency spans indications of use, administration modalities, dosage parameters, anticipated outcomes, follow-up strategies, and preparation methodologies. Despite the limited presence of review articles, the absence of a consensus to standardize preparation protocol based on platelet bio physiology and specific indications of use is glaring [1].

Wound healing is a multistep process in which cellular, immunological, and hormonal components work together to heal a wound. For the majority of patients, wound healing is unabated and without complications, but for many others, wound healing is not that straightforward. Studies of chronic wound fluid suggested a reduced level of growth factor and an increased level of proteases [1]. Chronic wounds may thus have an intrinsic molecular deformity that prevents them from healing. This provided a new treatment window by encouraging chronic wound healing by therapeutic manipulation of the required growth factors [2].

Platelets are the first cellular components to respond at the wound site during normal wound healing. Platelet activation causes the release of components that are required for wound healing. The wound-healing cascade can be broken down into the following four phases: hemostasis, inflammation, proliferation, and remodeling [3].

Within an hour of beginning of hemostasis, trapped platelets in the fibrin network release more than 95% of their pre-synthesised growth factors [4]. The released growth factors activate the wound-healing cascade by attracting and activating fibroblasts, endothelial cells, and macrophages, resulting in an inflammatory response, which is the second phase of wound healing [5]. Macrophages secrete cytokines, which cause fibroblasts and endothelial cells to flock to the wound site [6]. The proliferative phase follows, which is characterized by fibroblast proliferation and the production of mostly new collagen [7]. Several factors attract fibroblasts, the most prominent of which are transforming growth factor (TGF) and platelets-derived growth factor (PDGF). Granulation tissue is produced by growth factor-stimulated fibroblasts, which stimulate angiogenesis inside the granulation tissue [8]. Platelet-derived growth factors activate inflammatory cells and are crucial in the healing process’s early stages. They also encourage cell division and migration, as well as protein and extracellular matrix formation. Platelets can be concentrated to acquire a higher number of growth factors, which may help to speed up the slowed healing processes.

TGF and PDGF appear to be the most effective in vivo stimulators of proliferation, cellular differentiation, chemotaxis, and apoptosis in wound-healing cells [9]. The healing properties of PDGF have been exploited in the form of autologous PRP units, which have been used to improve healing in a variety of disciplines. For a better healing effect, a four-to-five-fold increase in platelet concentration is recommended. Graziani et al. propose that the optimal PRP concentration is at least 2.5 times the baseline, cautioning that exceeding this threshold might result in inhibitory effects [10].

The benefits of autologous PRP include increased tissue healing support, faster collagen mineralization in bone regeneration, early graft stability, cytokines, and growth factors, no risk of disease transmission, and improved osteoconduction [10]. The preparation protocols for platelet-rich plasma (PRP) exhibit significant variability, influenced by several critical factors. These encompass the platelet count in the donor, the use of a wide-bore needle for blood collection, the optimal blood volume for PRP extraction, and diverse methods for PRP procurement, such as open and closed systems. Additionally, the activation methods employed in the PRP preparation process warrant examination, including a comparative analysis of bovine thrombin versus autologous thrombin for PRP activation.

Further considerations involve the effects of varying concentrations of calcium chloride (2.5%, 5%, and 10%) on PRP activation, an evaluation of centrifugation protocols impacting the composition and quality of PRP, and scrutiny of methodologies for quality control during PRP preparation. Optimal centrifugation parameters and characteristics, including temperature control, warrant investigation, as does a comparative analysis of revolutions per minute (RPM) versus relative centrifugal force (RCF) in the context of centrifugation.

The types of centrifuges employed, varying frequencies of PRP injections, basic formulas relevant for calculations in PRP preparation, and an assessment of platelet purity versus platelet yield constitute essential components of this comprehensive examination. Finally, a valid optimal treatment schedule is a critical aspect that necessitates careful consideration in the pursuit of standardized and effective PRP protocols.

These variations in PRP products result from several influencing factors, including (i) diverse preparation methods involving manual techniques, the use of kits for component separation, and apheresis, where pure PRP is separated during blood collection, and the unused blood fraction is reinfused into the patient; (ii) variations in the amount, speed, and duration of centrifugation, determining the quantity of concentrated platelets and impacting their aggregation capacity in the final product; (iii) the presence or absence of leukocytes; (iv) the utilization of different anticoagulants such as citrate dextrose, sodium citrate, and heparin; and (v) the application or omission of agonists or platelet activators like calcium chloride, adenosine diphosphate (ADP), epinephrine, collagen, and thrombin.

The influence of anticoagulant and antiaggregating agents during PRP preparation, the number of spins during centrifugation, and a comparative assessment of single versus double centrifugation protocols also demand exploration. Moreover, PRP characteristics are influenced by patient-specific biological conditions, encompassing age, gender, concurrent diseases, hormone imbalances, blood disorders, and the use of medications such as anti-inflammatory drugs, acetylsalicylic acid, antibiotics, and various other pharmaceutical classes affecting platelet degranulation. This article studies the clinical outcome of nonhealing chronic ulcers treated with platelet-rich plasma prepared by single and double spin methods.

## Materials and methods

This study was undertaken to validate a protocol for PRP preparation by single and double spin methods and to correlate it with clinical outcomes of nonhealing chronic ulcers. This study included cases of nonhealing chronic ulcers (NHCU) after obtaining written informed consent from the patients and getting approval from the institution's human ethics committee.

The study population included patients from the outpatient or those admitted as inpatients for managing chronic wounds from 2018 to 2020. Inclusion criteria were adult patients of all sexes (male, female, and transgender) between the age group of 18 and 85 years with chronic nonhealing ulcers of etiologies such as diabetes, pressure ulcers, and leprosy ulcers. Exclusion criteria included patients on anticoagulant therapy, patients on aspirin and immunosuppressive medicine, patients with platelet counts <150×10^9^/L, presence of platelet dysfunction syndrome, bleeding disorders, and collagen vascular disease, radiation or chemotherapy within three months prior to PRP treatment, and pregnant and breast-feeding females. Data collection was done after taking a detailed history of all cases regarding the duration, mode of onset, progression, and associated symptoms.

Initially, 36 individuals were chosen for the study. Following dropouts, 22 individuals were ultimately kept in the research and added for analysis. Patients were randomized into two groups (SS and DS) by sealed envelope randomization, with 11 patients in each group. Eleven cases were chosen for the study with autologous platelet gel prepared by single spin, and 11 cases were chosen for the study with autologous platelet gel prepared by double spin. The total number of ulcers treated by the double spin method was 18, and the total number of ulcers treated by the single spin method was 13.

The following factors were kept constant for both PRP preparation methods, and the only variable factor was single spin versus double spin. The constant factors were the use of a wide-bore needle for blood collection, the optimal blood volume for PRP extraction, and closed systems for PRP preparation. Autologous thrombin was used for PRP activation in both preparation methods and concentrations of calcium chloride used was 2.5% for PRP activation. The anticoagulant used for both methods was acid citrate dextrose, the number of spins during centrifugation for the first spin, and time along with RPM and RCF were the same for both methods, the double spin method had a different time and speed of centrifugation for the second spin. The centrifugation protocol for the first spin was 3400 rpm for 10 min for both methods, and for the second spin, the centrifugation protocol was 1800 rpm for 9 min which was used for double spin method. Thus a comparative assessment of single versus double centrifugation protocols was done with the same treatment schedule for PRP prepared by both methods.

Nonhealing chronic ulcer (NHCU) was first cleaned with saline. Then, freshly prepared PRP was injected into the ulcer margin and base, applied over the ulcer, and the ulcer was dressed with sterile cotton gauze. The dressing was changed on the fifth day, 15th day, and 20th day, with infiltration of PRP every time. Results were observed on the 30th day from the first sitting. Fourteen patients were lost to follow-up and 22 patients completed the treatment protocol.

The assessment tools used were as follows: the surface area of ulcer was measured in square cm and the volume of the pressure ulcer was measured in milliliter. The percentage of healed ulcers was measured by subtracting the original ulcer area from the final ulcer area × 100/original ulcer area.

Statistical analysis was done by data collection in MS Excel (Redmond, WA: Microsoft Corp.) and analyzed using R software v4.2.0 (Vienna, Austria: R Foundation for Statistical Computing). For descriptive statistics, all the continuous variables such as size of the ulcer and volume of the ulcer were described as means and standard deviation or median and interquartile range. And, all the categorical variables, such as sex and completion status, were summated as frequencies and percentages.

## Results

The number of patients treated by double spin was 11, and the number of patients treated by single spin was 11. However, the number of ulcers in these patients varied; the total number of ulcers treated by double spin was 18, and the total number of ulcers treated by single spin was 13.

The mean age distribution in the single spin group was 39.00±10.99, and in the double spin group, 54.55±16.45, and the p-value was 0.027. The mean hemoglobin level in the single spin was 10.79±1.88, in the double spin 12.63±2.22, and p-value was 0.049. The platelet count was within the normal range for the participants. The study group had baseline platelet count varying from 1,50,000/mm^3^ to 4,23,000/mm^3^ with a maximum number of cases of 59% (n=135/22) having a platelet count within the range of 1,50,000. The single spin group showed a higher PRP thrombocyte count and a significant increase in thrombocyte count, compared to the double spin group, 2,50,000/mm^3^ (Table 1). The single spin group showed a higher PRP thrombocyte count and a significant increase in thrombocyte count compared to the double spin group.

**Table 1:** The surface area of nonhealing chronic ulcers.

Platelet count categories	No. of cases (n)	Percentage
1,50,000-2,50,000/mm^3^	13	59.09%
2,51,000-3,50,000/mm^3^	07	31.82%
>3,51,000/mm^3^	02	9.09%
Total	22	-

In the study group, the maximum platelet concentration achieved in PRP was 12.06 times of the baseline platelet count with mean concentration of platelets in PRP being 9.082 times the baseline platelet count. Maximum number of cases, 50% (n=11/22), had PRP with platelet concentration up to 5-10 times the baseline platelet. The single spin group showed a higher PRP thrombocyte count and a significant increase in thrombocyte count, compared to the double spin group. Platelet concentration of more than 10 times the baseline platelet count was achieved in 13.6% (n=03/22) cases (Table 2).

**Table 2:** Concentration of platelets in PRP. PRP: platelet-rich plasma

Concentration	No. of cases (n)	Percentage
4-5 times	11	50%
5-10 times	08	36.36%
>10 times	03	13.6%
TOTAL	22	100%

**Figure 1:**
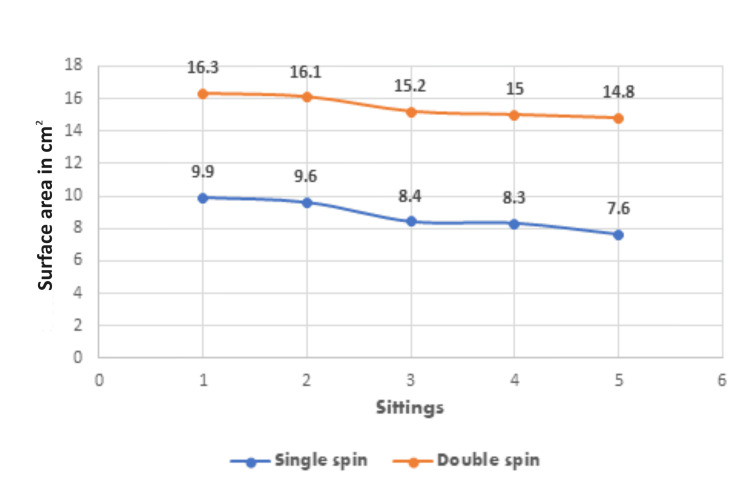
The comparison of surface area of nonhealing chronic ulcers.

The surface area of the ulcers was measured for all five sittings in both single spin and double spin PRP treatments. The mean area at all five sittings was compared using Wilcoxon rank sum test and the difference in the means of the two groups was found to be statistically significant with a p-value of 0.049. The mean and standard deviation of surface area at the first sitting in the single spin group was 16.27 (11.94) and the range was 5.00-52.25 as compared to the surface area in the double spin group, which was reported to be 9.87 (7.73) and the range was reported to be 1.00-22.75. In the final measurement, the surface area in double spin was 14.76 (11.54), the range was 5.00-48.76, and p-value was 0.020 compared to the surface area in single spin 7.56 (6.23) and range of 0.64-20.00 (Figure 1).

**Figure 2:**
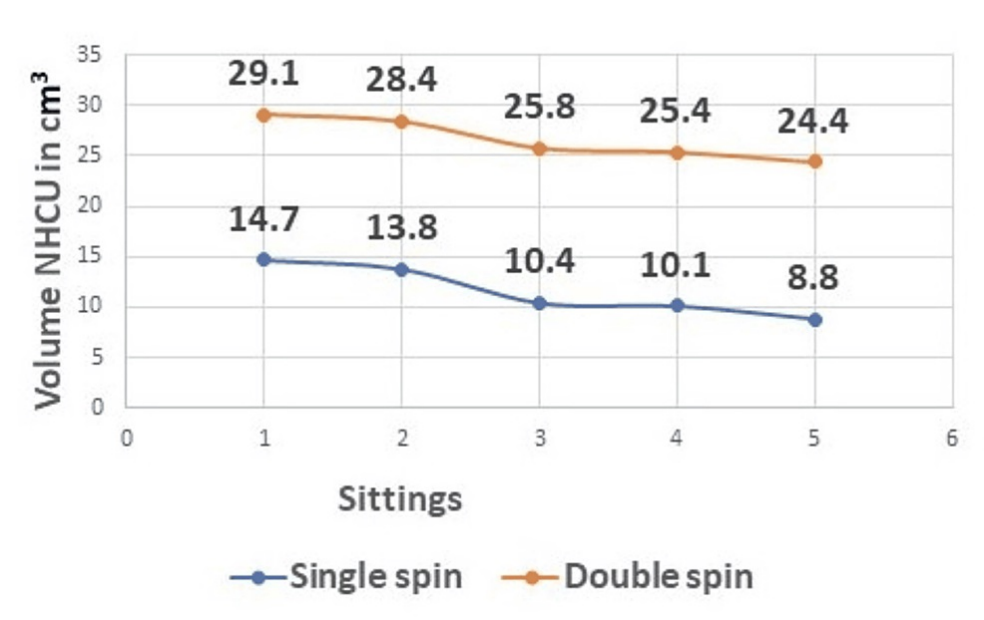
The volume of nonhealing chronic ulcer (NHCU).

In our study, we compared the ulcer volume at all five sittings. The mean and standard deviation of the volume of NHCU at the first sitting in the double spin group was 29.10 (34.15), and the range was 5.00-52.25 as compared to the mean and standard deviation of the volume of NHCU in the single spin group 14.72 (17.39) and range of 0.050-54.00 with a p-value of 0.047. In the final measurement, the mean volume in double spin was 24.38 (32.35) with a range of 2.50-146.28 compared to the single spin mean volume of 8.78 (11.07) with a range of 0.19-33.60 and p-value of 0.020. The means were compared using the Wilcoxon rank sum test, and the difference in the means of the two groups was found to be statistically significant with a p-value of <0.05 (Figure 2).

**Figure 3:**
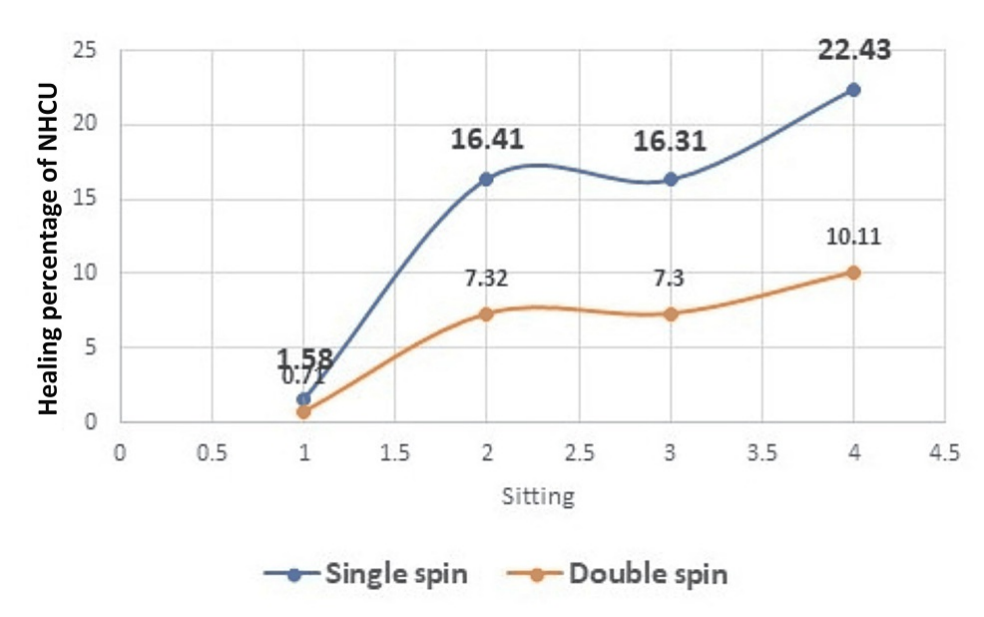
Healing percentage of NHCU by single spin and double spin PRP. NHCU: nonhealing chronic ulcer; PRP: platelet-rich plasma

**Figure 4:**
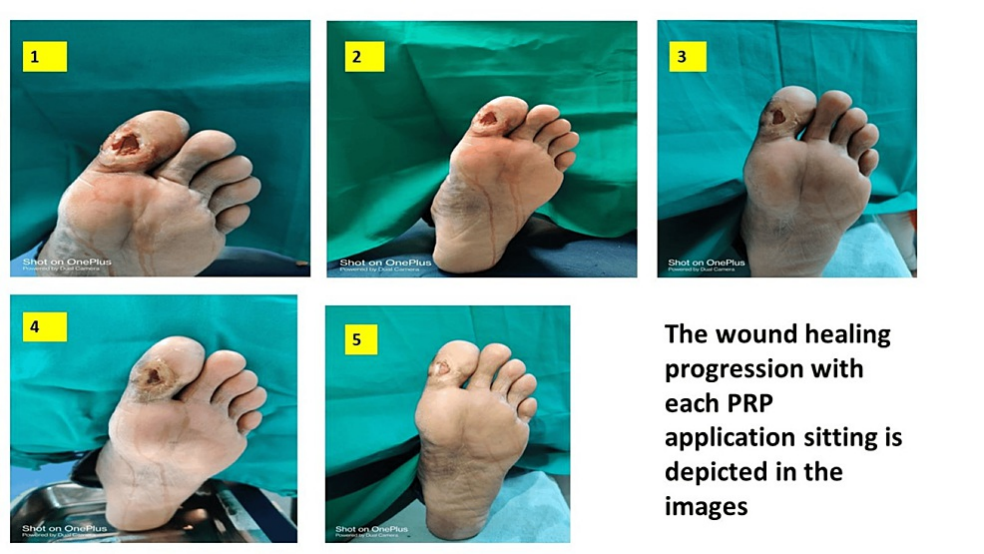
Wound healing with PRP application. PRP: platelet-rich plasma

The wound healing of the ulcer was calculated by subtracting the original ulcer area from the healed ulcer area. Percentage of healed ulcer = original ulcer area - final ulcer area × 100. The mean of healed area by single spin PRP was 22.43 (16.70) compared to the mean of healed area by double spin PRP which was 10.11 (12.03). The healed area of ulcers increased by both single spin PRP and double spin PRP treatment. However, a comparative analysis reveals a significantly superior outcome in the single spin PRP method concerning the percentage of healing area, volume, and overall percentage of healing in all sessions (Figure 3). Figure 4 shows the progression of ulcer healing with the single spin method of PRP treatment, including a reduction in ulcer size and overall improvement in healing with each PRP treatment session.

## Discussion

Nonhealing chronic ulcer (NHCU) poses a substantial threat, often leading to limb deformity, nonfunctionality, and, in severe cases, amputation. Despite the potential for prevention with proper care, patients with NHCU remain susceptible to persistent limb issues. Local wound management strategies involve debridement, cleansing, infection treatment, and dressing application. The utilization of platelet-rich plasma (PRP) in chronic ulcer treatment, supported by literature evidence, has demonstrated positive outcomes [11-15].

Our study evaluates the efficacy of locally applied PRP, prepared through two distinct methods (single spin and double spin), in the treatment of NHCU. The application of autologous PRP serves as a rich source of growth factors, accelerating the formation of granulation tissue during the early phases of healing. Previous research has highlighted the benefits of PRP in addressing various ulcer origins, such as diabetic ulcers, leprotic ulcers, and pressure ulcers.

Results from our study indicate notable improvements in both single spin and double spin PRP methods across various assessment parameters. However, a comparative analysis reveals a significantly superior outcome in the single spin PRP method concerning the percentage of healing area, volume, and overall percentage of healing in all sessions. This research contributes valuable insights into optimizing PRP treatment protocols for NHCU, offering clinicians evidence-based guidance for improved patient outcomes.

The observed variations in outcomes associated with platelet-rich plasma (PRP) therapies may be attributed to the absence of standardized parameters for PRP preparation, both in manual methods and automatic devices. Divergence in device specifications and blood collection protocols results in variability in the volume of patient-derived whole blood, centrifugal force applied during PRP preparation, number of centrifugation steps, and ultimately, the volume of PRP and the resultant platelet content. The inevitable consequence of variable platelet content is the diverse concentrations of platelet-derived growth factors.

Several factors influence PRP preparation and final platelet count, with the amount of whole blood withdrawn from the patient being paramount. Various methods, as documented in different studies, range from utilizing 10 mL of patient blood spun in a clinical centrifuge to employing a unit (400-500 mL) of blood processed through a cell separator for platelet concentration. In our study, a 100 mL volume yielded a sufficient PRP volume (20-30 mL) with a 4-5-fold or higher platelet enrichment following a double spin, aligning with recommendations from various authors.

In contrast, other studies demonstrated wide-ranging approaches, with blood volumes ranging from 8.5 mL to 60 mL, resulting in varied PRP volumes (0.5-10 mL). This disparity hampers effective comparison of PRP products across different clinical settings. To address this challenge, further large-scale studies are imperative to establish uniformity in PRP product volume, facilitating more accurate and meaningful comparisons across diverse clinical scenarios [16-20].

Lei et al. emphasize the superiority of acid-citrate-dextrose (ACD) over heparin and citrate in maintaining platelet structure integrity and preventing spontaneous platelet activation [21]. ACD-PRP has been shown to release more growth factors compared to heparin-PRP and citrate-PRP, emphasizing the crucial role of anticoagulant selection in PRP quality. Some protocols take additional steps to enhance platelet activation and ensure fibrin clot formation by combining PRP-Ca^2+^ preparation with exogenous thrombin, autologous thrombin, ethanol, or a negatively charged surface such as glass [11,21,22]. Understanding the impact of anticoagulant choice on PRP composition and quality is essential for refining PRP preparation protocols and optimizing therapeutic outcomes.

Ensuring the standardization of centrifugation speed and technique is imperative during platelet-rich plasma (PRP) preparation, as both variables significantly influence the final platelet concentration in PRP. Numerous authors have reported on the utilization of both single and double spin techniques at various centrifugation speeds. Arora et al. elucidate the heterogeneity of platelets and biomaterials when subjected to different g-forces and times. While the study does not propose the optimal PRP preparation method, it provides an in vitro estimation of the quantity of growth factors released from PRP prepared using these methods. Consequently, this study elucidates various factors affecting PRP quality, particularly highlighting that variations in both force (g) and time lead to cellular and growth factor concentration changes [23].

In our study, we conducted a comparative analysis of the single and double spin methods at a speed of 1800 rpm for 10 minutes and 3400 rpm for 9 minutes. This approach contributes valuable insights into the impact of specific centrifugation parameters on PRP composition, aiding in the development of standardized protocols for PRP preparation with potential implications for optimizing therapeutic efficacy.

Riboh et al. conducted a Cochrane review revealing comparable safety profiles in both leukocyte-rich and leukocyte-poor PRP formulations. While Dragoo et al. noted that leukocyte-rich PRP induces a more pronounced inflammatory effect than its leukocyte-poor counterpart, the ultimate impact on the healing process remains uncertain [24,25].

Platelet concentration stands out as a crucial variable affecting PRP product quality. Achieving consistent platelet concentrations is challenging due to differences in preparation techniques, whether automatic or manual. While automatic commercial kits claim to offer constant platelet concentrations, we endeavored to standardize the cost-effective manual PRP gel preparation method. This approach holds promise for future research in resource-constrained settings, such as India, where the widespread affordability of commercial kits is challenging. The platelet concentrations achieved in our present study align with previous investigations by Filardo et al. [26]. Notably, our study reveals a linear correlation (r=0.608) between platelet counts in whole blood and platelet-rich plasma (PRP), underscoring the predictive utility of whole blood platelet counts for PRP platelet counts.

Emphasizing the importance of platelet concentration in PRP preparation, our study observed PRP platelet concentrations ranging from five to 10 times the baseline platelet counts, with a mean concentration of 9.08 times the baseline counts. It is essential to recognize that achieving high platelet concentrations alone does not necessarily indicate a quality PRP product. Fitzpatrick et al. later suggested that PRP preparations should be enriched to a platelet concentration between four to five-fold the baseline counts, cautioning against overconcentrating platelets, as it may not further enhance wound healing [27].

The surface area of the NHCU was found to be significantly reduced following treatment with PRP with single spin and PRP with double spin. In the study conducted by Bhatia et al., the single spin method demonstrated a platelet concentration factor of 2.19, while the double spin method resulted in a reduction in platelet counts (platelet concentration factor=0.83). Our study also demonstrated similar findings, therefore it is safe to conclude that the single centrifugation method surpasses the double centrifugation method in preparing platelet-rich plasma, as indicated by the higher platelet concentration factor [28].

Improvement in ulcers was recorded in both groups in most of the parameters. Treatment with PRP prepared by the single spin method was found to result in better outcomes with respect to healing area and volume. Our study's findings contrast with the findings reported in the literature [29].

The findings of our study reaffirm the efficacy of PRP in the treatment of nonhealing chronic ulcer-related patients, and PRP prepared by the single spin method showed a better and more effective outcome in comparison to the double spin method. However, long-term study involving a larger sample size is warranted for further consolidation of our findings.

The limitation of our study was the small sample size. This study was conducted during the COVID-19 pandemic, which resulted in a reduced number of patients and limited follow-up. Additionally, the lack of a control group calls for further studies involving a larger sample size and long-term follow-up to further consolidate our findings. The power of a study is positively correlated with the sample size, which means that a larger sample size gives greater power.

## Conclusions

Improvement in ulcer healing was recorded in both groups in most of the parameters. Treatment with PRP prepared by the single spin method resulted in better outcomes with respect to healing area and volume. The single spin group showed a higher PRP thrombocyte count and a significant increase in thrombocyte count compared to the double spin group.

Our study's findings reaffirm PRP's efficacy in treating nonhealing chronic ulcer-related patients as seen in other studies. PRP prepared by the single spin method showed a better and more effective outcome than the double spin method. However, a long-term study involving a larger sample size is warranted for further consolidation of our findings as a larger sample size enables us to find smaller differences that are statistically significant, and the small sample size in our study has a low chance of detecting a difference between the two groups.
